# 2-Amino-4-(4-meth­oxy­phen­yl)-5-oxo-5,6,7,8-tetra­hydro-4*H*-chromene-3-carbonitrile

**DOI:** 10.1107/S1600536811043674

**Published:** 2011-10-29

**Authors:** Lingqian Kong, Xiuping Ju, Yan Qiao, Jidong Zhang, Zhiqing Gao

**Affiliations:** a Dongchang College, Liaocheng University, Liaocheng 250059, People’s Republic of China; b College of Chemistry and Chemical Engineering, Liaocheng University, Shandong 252059, People’s Republic of China

## Abstract

The title compound, C_17_H_16_N_2_O_3_, crystallizes with two independent mol­ecules in the asymmetric unit. In both mol­ecules, the fused cyclo­hexenone ring adopts a sofa conformation. In the crystal, N—H⋯N and N—H⋯O hydrogen bonds link the mol­ecules into corrugated layers parallel to the (101) plane.

## Related literature

For the crystal structures of related compounds, see: Nesterov *et al.* (2004 ▶); Wang & Zhu (2007 ▶). For applications of benzopyran derivatives, see: O’Callaghan *et al.* (1995 ▶).
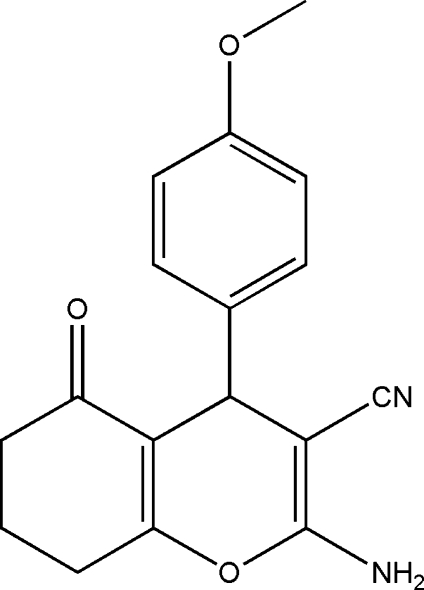


## Experimental

### 

#### Crystal data

C_17_H_16_N_2_O_3_
*M* *_r_* = 296.32
Monoclinic, 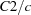

*a* = 31.973 (3) Å
*b* = 8.7750 (8) Å
*c* = 22.6861 (2) Å
β = 106.766 (1)°
*V* = 6094.4 (8) Å^3^
*Z* = 16
Mo *K*α radiation
μ = 0.09 mm^−1^
*T* = 298 K
0.43 × 0.42 × 0.38 mm

#### Data collection

Bruker SMART APEX CCD area-detector diffractometer
Absorption correction: multi-scan (*SADABS*; Sheldrick, 1996 ▶) *T*_min_ = 0.962, *T*_max_ = 0.967
14893 measured reflections
5361 independent reflections
2215 reflections with *I* > 2σ(*I*)
*R*_int_ = 0.066

#### Refinement

*R*[*F* ^2^ > 2σ(*F* ^2^)] = 0.052
*wR*(*F* ^2^) = 0.160
*S* = 1.01
5361 reflections
399 parameters
H-atom parameters constrained
Δρ_max_ = 0.23 e Å^−3^
Δρ_min_ = −0.23 e Å^−3^

### 

Data collection: *SMART* (Bruker, 2007 ▶); cell refinement: *SAINT* (Bruker, 2007 ▶); data reduction: *SAINT*; program(s) used to solve structure: *SHELXS97* (Sheldrick, 2008 ▶); program(s) used to refine structure: *SHELXL97* (Sheldrick, 2008 ▶); molecular graphics: *SHELXTL* (Sheldrick, 2008 ▶); software used to prepare material for publication: *SHELXTL*.

## Supplementary Material

Crystal structure: contains datablock(s) I, global. DOI: 10.1107/S1600536811043674/cv5173sup1.cif

Structure factors: contains datablock(s) I. DOI: 10.1107/S1600536811043674/cv5173Isup2.hkl

Supplementary material file. DOI: 10.1107/S1600536811043674/cv5173Isup3.cml

Additional supplementary materials:  crystallographic information; 3D view; checkCIF report

## Figures and Tables

**Table 1:** Hydrogen-bond geometry (Å, °)

*D*—H⋯*A*	*D*—H	H⋯*A*	*D*⋯*A*	*D*—H⋯*A*
N1—H1*A*⋯N4	0.86	2.19	3.035 (5)	168
N3—H3*AA*⋯N2	0.86	2.23	3.083 (5)	173
N1—H1*C*⋯O5^i^	0.86	2.03	2.877 (4)	167
N3—H3*BA*⋯O2^ii^	0.86	2.27	3.029 (4)	148

Symmetry codes: (i) 
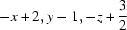
; (ii) 
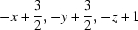
.

**Fig. 1.**
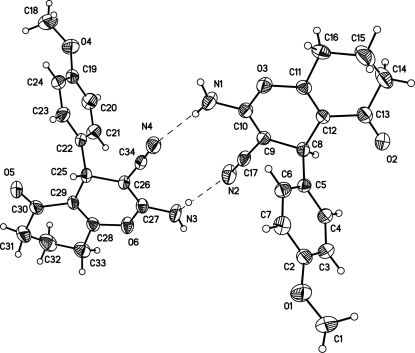
Two independent molecules in (I) with the atomic numbering scheme. The displacement ellipsoids are shown at the 30% probability level.The intermolecular hydrogen bonds represented by dashed lines.

## supplementary crystallographic information

### Comment

Benzopyran derivatives are useful starting materials for the preparation of polyheterocyclic compounds (O'Callaghan *et al.*, 1995), so much interest has still been paid to the design of polyfunctionalized substituted pyran derivatives (Wang & Zhu, 2007). We obtained the title compound, (I), and report here its crystal structure.

The asymmetric unit  of (I) contains two independent molecules (Fig. 1). The bond lengths and angles are normal and correspond to those observed in 2-amino- 4-(1-naphthyl)-5-oxo-5,6,7,8-tetrahydro-4H-chromene-3- carbonitrile (Nesterov *et al.*, 2004). In one independent molecule,  the pyran ring adopts a half-chair conformation. The  O3/C9/C10/C11/C12 plane forms an angle of 82.69 (11 )° with the benzene ring C2-C7. In another independent molecule,  the O6/C26/C27/C28/C29 plane and the benzen ring C19-C24 forms an angle of 89.24(11 )°. The triple bond lengths in nitrile groups are typical [N2≡C17 1.147 (4) Å, N4≡C34 1.142 (5)Å ].

In the crystal structure, intermolecular N—H···N and N—H···O hydrogen bonds (Table 1) link the molecules into corrugated layers parallel to (101) plane.

### Experimental

Malononitrile  (5 mmol), 1,3-cyclohexanedione (5 mmol),and 4-methoxybenzaldehyde (5 mmol)was dissolved in 20ml ethanol ml in a round-bottom flask. The mixture was warmed, with agitation, to 363 K over a period of  3 h. The resulting solution was cooled. Crystal of (I) suitable for X-ray diffraction analysis were obtained by slow evaporation.

### Refinement

All H atoms were placed in geometrically idealized positions (N-H 0.86 and C-H 0.93-0.98 Å ) and treated as riding on their parent atoms, with U_iso_(H) = 1.2U_eq_(C, N).

### Crystal data

**Table:**

C_17_H_16_N_2_O_3_	*F*(000) = 2496
*M**_r_* = 296.32	*D*_x_ = 1.292 Mg m^−^^3^
Monoclinic, *C*2/*c*	Mo *K*α radiation, λ = 0.71073 Å
*a* = 31.973 (3) Å	Cell parameters from 1799 reflections
*b* = 8.7750 (8) Å	θ = 2.5–21.2°
*c* = 22.6861 (2) Å	µ = 0.09 mm^−^^1^
β = 106.766 (1)°	*T* = 298 K
*V* = 6094.4 (8) Å^3^	Block, red
*Z* = 16	0.43 × 0.42 × 0.38 mm

### Data collection

**Table:**

Bruker SMART APEX CCD area-detector diffractometer	5361 independent reflections
Radiation source: fine-focus sealed tube	2215 reflections with *I* > 2σ(*I*)
graphite	*R*_int_ = 0.066
phi and ω scans	θ_max_ = 25.0°, θ_min_ = 1.3°
Absorption correction: multi-scan (*SADABS*; Sheldrick, 1996)	*h* = −38→32
*T*_min_ = 0.962, *T*_max_ = 0.967	*k* = −10→10
14893 measured reflections	*l* = −23→26

### Refinement

**Table:**

Refinement on *F*^2^	Primary atom site location: structure-invariant direct methods
Least-squares matrix: full	Secondary atom site location: difference Fourier map
*R*[*F*^2^ > 2σ(*F*^2^)] = 0.052	Hydrogen site location: inferred from neighbouring sites
*wR*(*F*^2^) = 0.160	H-atom parameters constrained
*S* = 1.01	*w* = 1/[σ^2^(*F*_o_^2^) + (0.0424*P*)^2^ + 5.9103*P*] where *P* = (*F*_o_^2^ + 2*F*_c_^2^)/3
5361 reflections	(Δ/σ)_max_ = 0.001
399 parameters	Δρ_max_ = 0.23 e Å^−^^3^
0 restraints	Δρ_min_ = −0.23 e Å^−^^3^

### Special details

**Table:**

Geometry. All esds (except the esd in the dihedral angle between two l.s. planes) are estimated using the full covariance matrix. The cell esds are taken into account individually in the estimation of esds in distances, angles and torsion angles; correlations between esds in cell parameters are only used when they are defined by crystal symmetry. An approximate (isotropic) treatment of cell esds is used for estimating esds involving l.s. planes.
Refinement. Refinement of F^2^ against ALL reflections. The weighted R-factor wR and goodness of fit S are based on F^2^, conventional R-factors R are based on F, with F set to zero for negative F^2^. The threshold expression of F^2^ > 2sigma(F^2^) is used only for calculating R-factors(gt) etc. and is not relevant to the choice of reflections for refinement. R-factors based on F^2^ are statistically about twice as large as those based on F, and R- factors based on ALL data will be even larger.

### Fractional atomic coordinates and isotropic or equivalent isotropic displacement parameters (Å^2^)

**Table:**

	*x*	*y*	*z*	*U*_iso_*/*U*_eq_	
N1	0.89836 (9)	0.5466 (4)	0.68379 (14)	0.0678 (11)	
H1A	0.9054	0.6339	0.6723	0.102*	
H1C	0.9166	0.4973	0.7128	0.102*	
N2	0.84053 (11)	0.7980 (4)	0.56300 (16)	0.0768 (12)	
O1	0.67083 (10)	0.8399 (4)	0.69415 (14)	0.0813 (10)	
O2	0.71575 (9)	0.2622 (3)	0.54178 (13)	0.0688 (9)	
O3	0.85498 (8)	0.3471 (3)	0.68076 (11)	0.0550 (7)	
C1	0.62873 (14)	0.8853 (5)	0.6583 (2)	0.0969 (17)	
H1D	0.6312	0.9454	0.6241	0.145*	
H1E	0.6151	0.9446	0.6831	0.145*	
H1F	0.6114	0.7965	0.6433	0.145*	
C2	0.69482 (13)	0.7493 (5)	0.6665 (2)	0.0566 (11)	
C3	0.68336 (12)	0.7138 (5)	0.6049 (2)	0.0592 (12)	
H3	0.6570	0.7481	0.5787	0.071*	
C4	0.71124 (12)	0.6267 (4)	0.58234 (18)	0.0528 (11)	
H4	0.7035	0.6046	0.5405	0.063*	
C5	0.75006 (11)	0.5715 (4)	0.61955 (17)	0.0415 (9)	
C6	0.76043 (12)	0.6052 (4)	0.68157 (18)	0.0523 (11)	
H6	0.7861	0.5669	0.7080	0.063*	
C7	0.73372 (13)	0.6939 (5)	0.70502 (18)	0.0582 (11)	
H7	0.7417	0.7168	0.7468	0.070*	
C8	0.78086 (11)	0.4813 (4)	0.59253 (16)	0.0415 (9)	
H8	0.7695	0.4864	0.5476	0.050*	
C9	0.82646 (11)	0.5476 (4)	0.61097 (16)	0.0422 (9)	
C10	0.85891 (12)	0.4876 (4)	0.65656 (17)	0.0476 (10)	
C11	0.81892 (12)	0.2610 (4)	0.65258 (17)	0.0462 (10)	
C12	0.78411 (11)	0.3167 (4)	0.61078 (16)	0.0425 (9)	
C13	0.74876 (14)	0.2132 (5)	0.57918 (19)	0.0543 (11)	
C14	0.75641 (16)	0.0466 (5)	0.5924 (2)	0.0837 (15)	
H14A	0.7283	−0.0032	0.5857	0.100*	
H14B	0.7701	0.0041	0.5631	0.100*	
C15	0.78339 (17)	0.0108 (5)	0.6542 (2)	0.0917 (16)	
H15A	0.7907	−0.0967	0.6561	0.110*	
H15B	0.7665	0.0289	0.6828	0.110*	
C16	0.82497 (13)	0.1015 (4)	0.67462 (19)	0.0615 (12)	
H16A	0.8354	0.1010	0.7192	0.074*	
H16B	0.8470	0.0533	0.6592	0.074*	
C17	0.83423 (12)	0.6861 (5)	0.58508 (17)	0.0511 (11)	
N3	0.87097 (9)	1.1189 (4)	0.53833 (14)	0.0658 (10)	
H3AA	0.8637	1.0304	0.5484	0.099*	
H3BA	0.8536	1.1678	0.5082	0.099*	
N4	0.92778 (11)	0.8666 (4)	0.66404 (16)	0.0669 (11)	
O4	1.12430 (10)	0.8579 (4)	0.61040 (15)	0.0882 (10)	
O5	1.03847 (9)	1.4277 (3)	0.70867 (15)	0.0820 (10)	
O6	0.91408 (8)	1.3208 (3)	0.54521 (11)	0.0597 (8)	
C18	1.15966 (15)	0.8117 (6)	0.6604 (2)	0.1049 (19)	
H18D	1.1737	0.8999	0.6824	0.157*	
H18E	1.1802	0.7554	0.6454	0.157*	
H18F	1.1491	0.7483	0.6875	0.157*	
C19	1.09169 (13)	0.9428 (5)	0.6230 (2)	0.0572 (11)	
C20	1.06204 (14)	1.0095 (5)	0.5733 (2)	0.0675 (13)	
H20A	1.0654	0.9990	0.5341	0.081*	
C21	1.02756 (12)	1.0913 (5)	0.58128 (18)	0.0574 (11)	
H21A	1.0076	1.1341	0.5471	0.069*	
C22	1.02170 (11)	1.1119 (4)	0.63865 (18)	0.0444 (10)	
C23	1.05165 (12)	1.0456 (5)	0.68757 (18)	0.0572 (11)	
H23A	1.0484	1.0577	0.7268	0.069*	
C24	1.08670 (13)	0.9607 (5)	0.6807 (2)	0.0604 (12)	
H24A	1.1065	0.9167	0.7148	0.072*	
C25	0.98294 (10)	1.1996 (4)	0.64763 (16)	0.0438 (10)	
H25A	0.9863	1.2041	0.6919	0.053*	
C26	0.93989 (11)	1.1221 (4)	0.61708 (16)	0.0433 (10)	
C27	0.90915 (12)	1.1805 (4)	0.56950 (17)	0.0467 (10)	
C28	0.94907 (12)	1.4101 (4)	0.57640 (19)	0.0512 (11)	
C29	0.98084 (11)	1.3592 (4)	0.62437 (18)	0.0476 (10)	
C30	1.01224 (13)	1.4693 (5)	0.6601 (2)	0.0612 (12)	
C31	1.01001 (15)	1.6301 (5)	0.6387 (3)	0.0897 (17)	
H31C	1.0394	1.6711	0.6487	0.108*	
H31D	0.9938	1.6896	0.6607	0.108*	
C32	0.98876 (16)	1.6474 (5)	0.5708 (2)	0.0880 (16)	
H32C	1.0080	1.6069	0.5485	0.106*	
H32D	0.9843	1.7549	0.5609	0.106*	
C33	0.94531 (14)	1.5655 (5)	0.5503 (2)	0.0719 (13)	
H33C	0.9236	1.6224	0.5636	0.086*	
H33D	0.9357	1.5596	0.5057	0.086*	
C34	0.93244 (11)	0.9796 (5)	0.64178 (18)	0.0477 (10)	

### Atomic displacement parameters (Å^2^)

**Table:**

	*U*^11^	*U*^22^	*U*^33^	*U*^12^	*U*^13^	*U*^23^
N1	0.045 (2)	0.074 (3)	0.072 (2)	−0.0108 (19)	−0.0044 (17)	0.026 (2)
N2	0.073 (3)	0.065 (3)	0.073 (3)	−0.023 (2)	−0.0094 (19)	0.032 (2)
O1	0.067 (2)	0.089 (2)	0.095 (2)	0.0112 (19)	0.0345 (18)	−0.0176 (19)
O2	0.0532 (18)	0.064 (2)	0.080 (2)	−0.0059 (16)	0.0051 (15)	−0.0098 (17)
O3	0.0483 (16)	0.0512 (19)	0.0579 (18)	−0.0022 (14)	0.0035 (13)	0.0201 (14)
C1	0.066 (3)	0.088 (4)	0.141 (5)	0.019 (3)	0.037 (3)	−0.015 (4)
C2	0.050 (3)	0.049 (3)	0.077 (3)	−0.004 (2)	0.029 (2)	−0.010 (2)
C3	0.046 (2)	0.058 (3)	0.067 (3)	0.007 (2)	0.006 (2)	0.000 (2)
C4	0.048 (2)	0.051 (3)	0.053 (3)	0.004 (2)	0.004 (2)	0.000 (2)
C5	0.038 (2)	0.038 (2)	0.047 (2)	0.0008 (18)	0.0085 (18)	0.0051 (19)
C6	0.051 (2)	0.055 (3)	0.047 (3)	0.005 (2)	0.008 (2)	0.006 (2)
C7	0.060 (3)	0.068 (3)	0.050 (3)	−0.005 (2)	0.020 (2)	0.000 (2)
C8	0.044 (2)	0.037 (2)	0.039 (2)	−0.0047 (18)	0.0049 (17)	0.0072 (18)
C9	0.042 (2)	0.039 (2)	0.045 (2)	−0.0007 (19)	0.0113 (18)	0.0102 (19)
C10	0.045 (2)	0.044 (3)	0.053 (3)	−0.005 (2)	0.012 (2)	0.010 (2)
C11	0.050 (2)	0.039 (3)	0.052 (3)	0.001 (2)	0.018 (2)	0.007 (2)
C12	0.045 (2)	0.037 (2)	0.045 (2)	−0.0005 (19)	0.0115 (19)	−0.0004 (19)
C13	0.057 (3)	0.050 (3)	0.059 (3)	−0.004 (2)	0.022 (2)	−0.006 (2)
C14	0.111 (4)	0.047 (3)	0.085 (4)	−0.020 (3)	0.015 (3)	0.001 (3)
C15	0.115 (4)	0.047 (3)	0.110 (5)	−0.012 (3)	0.028 (3)	0.010 (3)
C16	0.073 (3)	0.040 (3)	0.069 (3)	0.010 (2)	0.018 (2)	0.015 (2)
C17	0.043 (2)	0.054 (3)	0.047 (3)	−0.005 (2)	−0.0022 (19)	0.011 (2)
N3	0.051 (2)	0.070 (3)	0.060 (2)	−0.0189 (18)	−0.0100 (17)	0.0106 (19)
N4	0.063 (2)	0.055 (3)	0.073 (3)	−0.009 (2)	0.0033 (19)	0.011 (2)
O4	0.067 (2)	0.090 (3)	0.105 (3)	0.0193 (19)	0.0222 (19)	−0.021 (2)
O5	0.0555 (19)	0.077 (2)	0.098 (3)	−0.0100 (17)	−0.0034 (17)	−0.035 (2)
O6	0.0624 (18)	0.0550 (19)	0.0526 (18)	−0.0159 (15)	0.0021 (13)	0.0090 (15)
C18	0.066 (3)	0.096 (4)	0.147 (5)	0.021 (3)	0.024 (3)	0.001 (4)
C19	0.046 (3)	0.055 (3)	0.069 (3)	−0.004 (2)	0.016 (2)	−0.013 (2)
C20	0.065 (3)	0.087 (4)	0.053 (3)	0.003 (3)	0.022 (2)	−0.011 (3)
C21	0.054 (3)	0.072 (3)	0.047 (3)	0.004 (2)	0.015 (2)	−0.003 (2)
C22	0.036 (2)	0.046 (3)	0.048 (3)	−0.0049 (19)	0.0076 (19)	−0.004 (2)
C23	0.053 (3)	0.065 (3)	0.048 (3)	0.002 (2)	0.007 (2)	−0.002 (2)
C24	0.057 (3)	0.054 (3)	0.060 (3)	0.005 (2)	0.001 (2)	0.004 (2)
C25	0.039 (2)	0.050 (3)	0.041 (2)	−0.0093 (19)	0.0086 (17)	−0.0094 (19)
C26	0.038 (2)	0.046 (3)	0.043 (2)	−0.0060 (19)	0.0068 (18)	−0.003 (2)
C27	0.048 (2)	0.047 (3)	0.044 (3)	−0.010 (2)	0.012 (2)	0.001 (2)
C28	0.051 (3)	0.041 (3)	0.064 (3)	−0.009 (2)	0.021 (2)	−0.004 (2)
C29	0.040 (2)	0.046 (3)	0.057 (3)	−0.009 (2)	0.014 (2)	−0.013 (2)
C30	0.047 (3)	0.055 (3)	0.086 (4)	−0.005 (2)	0.025 (2)	−0.022 (3)
C31	0.074 (3)	0.054 (4)	0.137 (5)	−0.019 (3)	0.024 (3)	−0.024 (3)
C32	0.102 (4)	0.056 (3)	0.117 (5)	−0.016 (3)	0.049 (3)	−0.001 (3)
C33	0.084 (3)	0.052 (3)	0.082 (3)	−0.004 (3)	0.028 (3)	0.009 (3)
C34	0.036 (2)	0.051 (3)	0.049 (3)	−0.004 (2)	0.0026 (18)	−0.002 (2)

### Geometric parameters (Å, °)

**Table:**

N1—C10	1.338 (4)	N3—C27	1.336 (4)
N1—H1A	0.8600	N3—H3AA	0.8600
N1—H1C	0.8600	N3—H3BA	0.8600
N2—C17	1.147 (4)	N4—C34	1.142 (5)
O1—C2	1.376 (4)	O4—C19	1.377 (4)
O1—C1	1.414 (5)	O4—C18	1.411 (5)
O2—C13	1.225 (4)	O5—C30	1.231 (5)
O3—C10	1.370 (4)	O6—C27	1.376 (4)
O3—C11	1.373 (4)	O6—C28	1.382 (4)
C1—H1D	0.9600	C18—H18D	0.9600
C1—H1E	0.9600	C18—H18E	0.9600
C1—H1F	0.9600	C18—H18F	0.9600
C2—C3	1.373 (5)	C19—C24	1.373 (5)
C2—C7	1.386 (5)	C19—C20	1.377 (5)
C3—C4	1.380 (5)	C20—C21	1.371 (5)
C3—H3	0.9300	C20—H20A	0.9300
C4—C5	1.372 (4)	C21—C22	1.379 (5)
C4—H4	0.9300	C21—H21A	0.9300
C5—C6	1.381 (5)	C22—C23	1.369 (5)
C5—C8	1.523 (5)	C22—C25	1.521 (5)
C6—C7	1.371 (5)	C23—C24	1.391 (5)
C6—H6	0.9300	C23—H23A	0.9300
C7—H7	0.9300	C24—H24A	0.9300
C8—C12	1.498 (5)	C25—C29	1.492 (5)
C8—C9	1.513 (4)	C25—C26	1.512 (4)
C8—H8	0.9800	C25—H25A	0.9800
C9—C10	1.343 (5)	C26—C27	1.335 (5)
C9—C17	1.402 (5)	C26—C34	1.418 (5)
C11—C12	1.330 (4)	C28—C29	1.333 (5)
C11—C16	1.480 (5)	C28—C33	1.478 (5)
C12—C13	1.466 (5)	C29—C30	1.459 (5)
C13—C14	1.498 (5)	C30—C31	1.488 (6)
C14—C15	1.453 (6)	C31—C32	1.500 (6)
C14—H14A	0.9700	C31—H31C	0.9700
C14—H14B	0.9700	C31—H31D	0.9700
C15—C16	1.503 (5)	C32—C33	1.514 (5)
C15—H15A	0.9700	C32—H32C	0.9700
C15—H15B	0.9700	C32—H32D	0.9700
C16—H16A	0.9700	C33—H33C	0.9700
C16—H16B	0.9700	C33—H33D	0.9700
			
C10—N1—H1A	120.0	C27—N3—H3AA	120.0
C10—N1—H1C	120.0	C27—N3—H3BA	120.0
H1A—N1—H1C	120.0	H3AA—N3—H3BA	120.0
C2—O1—C1	117.6 (4)	C19—O4—C18	117.9 (4)
C10—O3—C11	118.1 (3)	C27—O6—C28	118.1 (3)
O1—C1—H1D	109.5	O4—C18—H18D	109.5
O1—C1—H1E	109.5	O4—C18—H18E	109.5
H1D—C1—H1E	109.5	H18D—C18—H18E	109.5
O1—C1—H1F	109.5	O4—C18—H18F	109.5
H1D—C1—H1F	109.5	H18D—C18—H18F	109.5
H1E—C1—H1F	109.5	H18E—C18—H18F	109.5
C3—C2—O1	125.1 (4)	C24—C19—O4	124.3 (4)
C3—C2—C7	119.3 (4)	C24—C19—C20	119.4 (4)
O1—C2—C7	115.6 (4)	O4—C19—C20	116.3 (4)
C2—C3—C4	119.4 (4)	C21—C20—C19	120.3 (4)
C2—C3—H3	120.3	C21—C20—H20A	119.9
C4—C3—H3	120.3	C19—C20—H20A	119.9
C5—C4—C3	122.3 (4)	C20—C21—C22	121.8 (4)
C5—C4—H4	118.9	C20—C21—H21A	119.1
C3—C4—H4	118.9	C22—C21—H21A	119.1
C4—C5—C6	117.4 (4)	C23—C22—C21	117.1 (4)
C4—C5—C8	120.7 (4)	C23—C22—C25	120.8 (4)
C6—C5—C8	121.9 (3)	C21—C22—C25	122.0 (3)
C7—C6—C5	121.6 (4)	C22—C23—C24	122.3 (4)
C7—C6—H6	119.2	C22—C23—H23A	118.8
C5—C6—H6	119.2	C24—C23—H23A	118.8
C6—C7—C2	120.0 (4)	C19—C24—C23	119.2 (4)
C6—C7—H7	120.0	C19—C24—H24A	120.4
C2—C7—H7	120.0	C23—C24—H24A	120.4
C12—C8—C9	108.1 (3)	C29—C25—C26	108.5 (3)
C12—C8—C5	113.0 (3)	C29—C25—C22	112.5 (3)
C9—C8—C5	111.8 (3)	C26—C25—C22	112.3 (3)
C12—C8—H8	107.9	C29—C25—H25A	107.7
C9—C8—H8	107.9	C26—C25—H25A	107.7
C5—C8—H8	107.9	C22—C25—H25A	107.7
C10—C9—C17	118.1 (3)	C27—C26—C34	119.3 (3)
C10—C9—C8	122.1 (3)	C27—C26—C25	123.9 (4)
C17—C9—C8	119.4 (3)	C34—C26—C25	116.7 (3)
N1—C10—C9	128.3 (4)	C26—C27—N3	128.3 (4)
N1—C10—O3	110.3 (3)	C26—C27—O6	121.7 (3)
C9—C10—O3	121.4 (3)	N3—C27—O6	110.0 (3)
C12—C11—O3	123.2 (3)	C29—C28—O6	122.8 (4)
C12—C11—C16	126.1 (4)	C29—C28—C33	126.2 (4)
O3—C11—C16	110.7 (3)	O6—C28—C33	111.0 (4)
C11—C12—C13	119.3 (4)	C28—C29—C30	118.2 (4)
C11—C12—C8	121.9 (3)	C28—C29—C25	123.3 (3)
C13—C12—C8	118.7 (3)	C30—C29—C25	118.1 (4)
O2—C13—C12	120.7 (4)	O5—C30—C29	118.9 (4)
O2—C13—C14	122.6 (4)	O5—C30—C31	122.0 (4)
C12—C13—C14	116.6 (4)	C29—C30—C31	119.0 (4)
C15—C14—C13	114.9 (4)	C30—C31—C32	113.4 (4)
C15—C14—H14A	108.6	C30—C31—H31C	108.9
C13—C14—H14A	108.6	C32—C31—H31C	108.9
C15—C14—H14B	108.6	C30—C31—H31D	108.9
C13—C14—H14B	108.6	C32—C31—H31D	108.9
H14A—C14—H14B	107.5	H31C—C31—H31D	107.7
C14—C15—C16	114.2 (4)	C31—C32—C33	111.8 (4)
C14—C15—H15A	108.7	C31—C32—H32C	109.3
C16—C15—H15A	108.7	C33—C32—H32C	109.3
C14—C15—H15B	108.7	C31—C32—H32D	109.3
C16—C15—H15B	108.7	C33—C32—H32D	109.3
H15A—C15—H15B	107.6	H32C—C32—H32D	107.9
C11—C16—C15	112.2 (3)	C28—C33—C32	110.7 (4)
C11—C16—H16A	109.2	C28—C33—H33C	109.5
C15—C16—H16A	109.2	C32—C33—H33C	109.5
C11—C16—H16B	109.2	C28—C33—H33D	109.5
C15—C16—H16B	109.2	C32—C33—H33D	109.5
H16A—C16—H16B	107.9	H33C—C33—H33D	108.1
N2—C17—C9	178.9 (5)	N4—C34—C26	177.0 (4)

### Hydrogen-bond geometry (Å, °)

**Table:**

*D*—H···*A*	*D*—H	H···*A*	*D*···*A*	*D*—H···*A*
N1—H1A···N4	0.86	2.19	3.035 (5)	168.
N3—H3AA···N2	0.86	2.23	3.083 (5)	173.
N1—H1C···O5^i^	0.86	2.03	2.877 (4)	167.
N3—H3BA···O2^ii^	0.86	2.27	3.029 (4)	148.

Symmetry codes: (i) −*x*+2, *y*−1, −*z*+3/2; (ii) −*x*+3/2, −*y*+3/2, −*z*+1.
